# Levers for Language Growth: Characteristics and Predictors of Language Trajectories between 4 and 7 Years

**DOI:** 10.1371/journal.pone.0134251

**Published:** 2015-08-04

**Authors:** Cristina McKean, Fiona K. Mensah, Patricia Eadie, Edith L. Bavin, Lesley Bretherton, Eileen Cini, Sheena Reilly

**Affiliations:** 1 Newcastle University, Newcastle upon Tyne, United Kingdom; 2 Murdoch Children’s Research Institute, Melbourne, Australia; 3 University of Melbourne, Melbourne, Australia; 4 La Trobe University, Melbourne, Australia; 5 The Royal Children’s Hospital, Melbourne, Australia; 6 Menzies Health Institute Queensland, Queensland, Australia; University of California, San Francisco, UNITED STATES

## Abstract

**Background:**

Evidence is required as to when and where to focus resources to achieve the greatest gains for children’s language development. Key to these decisions is the understanding of individual differences in children’s language trajectories and the predictors of those differences. To determine optimal timing we must understand if and when children’s relative language abilities become fixed. To determine where to focus effort we must identify mutable factors, that is those with the potential to be changed through interventions, which are associated with significant differences in children’s language scores and rate of progress.

**Methods:**

Uniquely this study examined individual differences in language growth trajectories in a population sample of children between 4 and 7 years using the multilevel model for change. The influence of predictors, grouped with respect to their mutability and their proximity to the child (least-mutable, mutable-distal, mutable-proximal), were estimated.

**Results:**

A significant degree of variability in rate of progress between 4 and 7 years was evident, much of which was systematically associated with mutable-proximal factors, that is, those factors with evidence that they are modifiable through interventions with the child or family, such as shared book reading, TV viewing and number of books in the home. Mutable-distal factors, such as family income, family literacy and neighbourhood disadvantage, hypothesised to be modifiable through social policy, were important predictors of language abilities at 4 years.

**Conclusions:**

Potential levers for language interventions lie in the child’s home learning environment from birth to age 4. However, the role of a family’s material and cultural capital must not be ignored, nor should the potential for growth into the school years. Early Years services should acknowledge the effects of multiple, cascading and cumulative risks and seek to promote child language development through the aggregation of marginal gains in the pre-school years and beyond.

## Introduction

Well-developed language abilities enable children to negotiate the social-relational demands of school life and provide the foundational knowledge upon which literacy and other academic skills are built [1]. Without such skills children are at increased risk of literacy difficulties [2] academic failure [3,4], social and emotional difficulties [5] and, as adults, of unemployment [6] and poor mental health [7]

Between 7–16% of 5 year olds have poor language development, that is falling 1–1.25 SD below the mean on norm-referenced language tests, with higher rates reported in more socially disadvantaged groups [8,9]. In recent years, the importance of promoting oral language development has been recognised in policy for Early Years provision internationally [10,11]. However as children transition into more formal schooling, this focus recedes. If potential remains to narrow the gap for children with poor language as they transition into school this may be an opportunity lost.

### Language Trajectories

Longitudinal studies suggest that in the pre-school years language development is highly variable, with ‘natural resolution’ of poor language occurring spontaneously for many children [12–14] but those children who reach 5 years with poor language are unlikely to ever catch up [3,15–17]. However, recent longitudinal studies of population cohorts suggest that the degree of fluidity and heterogeneity that exists in child language pathways has been underestimated [12–14,18].

The nature of language *trajectories* is poorly understood. Only five *population* studies of child language have considered the nature of language *growth* [18–22]. Two of these focussed on the crucial period of transition into formal schooling between 4 and 7 years but based their investigation on receptive vocabulary (i.e. knowledge of the from and meaning of individual words) [18,22]. Taylor, Christenson (18) explored the effect of child, maternal and family risk factors on vocabulary development from 4 to 8 years. This breadth of measurement aligns with current models of language development that acknowledge the influence of multiple and interacting influences on the language acquisition process. Similar to many other epidemiological samples, Taylor et al found that a range of child, maternal and family risks, predicted receptive vocabulary development at age 4. Vocabulary growth between 4 and 8 however was predicted by a much smaller number of factors. Speaking English as a second language (ESL), low school readiness and maternal mental health distress were all associated with lower scores at age 4 but also with ‘catch-up’ growth, although this was not sufficient to close the gap by 8 years. Furthermore children living in areas of socio-economic disadvantage did not have significantly poorer vocabulary scores at age 4 but did have lower rates of vocabulary growth between 4 and 8 years than their more advantaged peers.

‘Language’ is a complex developmental skill comprised not only of vocabulary knowledge but also grammatical morphology (i.e. markers which signal grammatical properties such as plurality or tense) and syntax (i.e. word order in sentences which, in conjunction with grammatical morphology, signals the nature of the relationships between components within sentences). Although receptive vocabulary has been highly correlated with broader language abilities cross-sectionally [23] the shape and rate of its developmental trajectory may not be representative of growth in syntax and morphology [24] since sensitive or optimal periods for development differ between language components [25]. Thus there may be greater variability in children’s growth rates during the optimal period for one component, and, conversely, variance in growth will decrease when a child’s relative ability in that component reaches maturation. Further research is therefore required to uncover the nature of individual differences in the broader language trajectory.

### Levers of language growth

Language development is the result of complex interactions between the child’s biological make-up, their family, school and community and the social and cultural context [11]. These interactions are hypothesised to involve cascading effects wherein distal factors, (those temporally or spatially distant from the child) indirectly affect child outcomes through their influence on more proximal factors which directly affect the child [26]. In public health, preventative interventions involve the removal or reduction of risk factors and/or the promotion of protective factors [27]. Preventing or ameliorating poor language development could, therefore, involve targeting any or all of these factors. Evidence is required as to where to focus effort, and hence resources, in order to gain maximal effect. Key to determining whether a factor should be targeted would be understanding both the scale of effect which it might yield, and also the degree to which it has the capacity to be changed; that is, its ‘mutability’. Factors vary in their mutability and hence their suitability as targets for preventative interventions, from least mutable, such as gender, which may confer risk but would not be a candidate for intervention, to most mutable, such as a family’s access to developmentally appropriate toys, which could readily be addressed through intervention. This study has two main aims. The first is to identify factors that are potentially modifiable by interventions that could reduce children’s risk of poor language development up to the age of 4 years and of poor language growth between 4 and 7 years. The second is to quantify the amount of variance these factors explain in order to estimate the potential gains that might be achieved.

Clearly factors fall on a continuum of how amenable they may be to change from most to least mutable and characterisation of risks as mutable or otherwise is not always simple. For the purposes of this study we developed a conceptual framework in which we grouped factors into three groups. ‘Least-mutable’ being those risks which are resistant to modification or inappropriate choices for intervention, ‘mutable-proximal’ as those which may be modified by direct interventions with the child or family, and ‘mutable-distal’, as those with indirect effects, which would require interventions delivered at a population level, through social policy rather than with individual families or schools. This framework allows us to build a series of models in a systematic and theoretically motivated sequence [28], and allows us to estimate the variability explained by each category of predictors in addition to the effects of the other categories.

This study is unique in exploring individual differences in children’s language abilities at 4 years and the *rate at which their language grows* between 4 and 7 years. By examining individual differences in growth after the age of 4 years and identifying mutable predictors of both language ability at 4 years and rate of progress, this paper seeks to inform decisions regarding where and when to focus resources to achieve the greatest gains for children’s language outcomes. To that end we ask the following research questions:

To what extent are children’s language trajectories fixed by age 4 and is there heterogeneity in children’s rate of progress between 4 and 7 years?

1. Which factors predict a child’s language at age 4 and which predict their rate of progress between 4 and 7?
2. How much of the variability in language abilities at age 4 and children’s rate of progress between 4 and 7 years is explained by mutable factors, either proximal or distal

## Materials and Method

### Participants and sampling

The participants were recruited into the Early Language in Victoria Study (ELVS), a community ascertained, prospective observational longitudinal study. Infants (mean age 8 months), were recruited in 2003–4 in metropolitan Melbourne, Victoria, Australia using a sampling frame stratified by the Australian Index of Social Disadvantage (Socio-economic Indexes for Areas—SEIFA) [29]. Data included in this paper were collected in 8 waves, from 8 months to 7 years. At baseline, 1910 children were recruited (50.5% boys), excluding children with serious disabilities or developmental delays (e.g. Down syndrome) and parents who did not speak and/or understand English. Further details are reported in Reilly et al. [30].

#### Ethics statement

Ethical approval was obtained from the Royal Childrens Hospital (#23018 and #27078) and La Trobe University, Human Ethics Committee (#03–32). All parents provided written, informed consent.

#### Attrition

The analyses were conducted on the subset of children who had completed language assessments at 4, 5 and 7 years. Children with complete (n = 883) and incomplete (n = 1027) outcome data, were compared. The groups were not significantly different with respect to gender (*p* = .1) but those with incomplete data had higher levels of social disadvantage (*p* < .001), lower language (4 years: *p* < .001, 5 years: *p* < .01, and 7 years: *p* < .001) and lower IQ (4 years: *p* < .001). This sample is more advantaged than the Australian population and so provides a conservative estimate of the effects of social disadvantage and associated factors in the wider population.

### Language Measures

Language was measured using direct assessment by trained research assistants, using the Australian adaptation of the Clinical Evaluation of Language Fundamentals-Preschool, Second Edition (CELF-P2) [31] at age 4 and the CELF-4 Australian Standardisation at 5 and 7 years [32]. To ensure that differences between the tests did not affect estimates of change over time, the CELF ‘core language’ raw scores were scaled to a mean 0 and *SD* of 1 (z scores) at each time point from for the sample of 883 children. The CELF is an omnibus language measure and tests children’s abilities to use and understand syntax (word order rules), semantics (word and sentence meanings) and grammatical morphology (markers of grammatical relationships such as plural—s and past tense—ed).

### Predictor Measures

Predictors were derived from direct assessment of non-verbal ability at 4 and parent report at 8 months, 1, 2, 3, and 4 years. Previous population studies were reviewed and potential risk and protective factors for language or vocabulary outcomes were identified in children between 4 and 7 years [13,15,19,33–37]. The final set of 22 predictors were chosen after preliminary analyses (see below), and were categorised as either ‘least mutable’, ‘mutable-distal’ or ‘mutable-proximal’.

#### Least-mutable factors

For the purposes of this study we considered factors to fall into the ‘least mutable’ category where the risks cannot be removed through the action of an intervention, either at the level of social policy or through an intervention delivered directly with the child or family. Factors were categorized as least- mutable if they fulfilled one or more of the following criteria: 1) cannot be modified through intervention as the factor is biologically driven (e.g. family history, gender); 2) comorbid diagnoses such as ADHD, ASD or Learning Disability whose symptoms can be ameliorated to a degree but which cannot be removed entirely 3) evidence for high levels of stability over development (e.g. Temperament); and 4) it would be unethical or impracticable to target in an intervention (e.g. exposure to non-dominant language in the home (see Table 1)).

**Table 1:** Least-mutable predictors: measures, age of measurement, derivation and criteria and evidence for categorisation.

Predictor	Age	Measure(s)	Derivation	Criteria	Evidence of stability over development*
Gender	8 months	Parental report		1	
Low birth weight	8 months	Parental report	Dichotomous variable categorising children as low (>2500g) or typical (≥ 2500g) birth weight	1	
Non-verbal IQ	4 years	Kaufman Brief Intelligence Test 2^nd^ Edition (KBIT-2)^a^	Kaufman Brief Intelligence Test Non-verbal standard scores were converted into quintiles (Q) based on the study sample (Q1 (highest standard scores) 121–146; Q2 112–118; Q3 103–110; Q4 97–101; Q5 (lowest standard scores) 55–97).	3	Moffitt TE, Caspi A, Harkness AR, Silva PA. The natural history of change in intellectual performance: Who changes? How much? Is it meaningful?. Journal of Child Psychology and Psychiatry. 1993;3(455–506).
Family history of speech and language difficulties	8 months	Parental report	Family history was coded as positive if the child's father, mother or siblings was reported to have either “been late to talk”, “had ongoing problems with speech or language during childhood", “had problems with stuttering”, or “had problems learning to read”	1	
Developmental disorder	All data waves		A dichotomous variable was derived categorising children as with or without a diagnosis of a developmental disorder if they met the criteria for any of the following as defined below: ADHD, Learning Difficulties, Developmental Delay or Autism Spectrum Disorder (ASD)	2	
		Parental report of diagnosis	- ADHD, Learning Difficulty and Developmental Delay were measured through parental report of a diagnosis and categorised as positive if reported at any data wave.		
		Parental interview	- ASD status was validated through parental interview by a Clinical Psychologist		
Shy/Approach-withdrawal	4 years	Australian Temperament Scale (ATS)^b^	Dichotomised as Shy/high approach-withdrawal (score ≥ 3.67) versus not shy/typical approach-withdrawal (score < 3.67). equivalent to 1 SD above the mean or higher (Prior et al 1989)	3	Pedlow R, Sanson A, Prior M, Oberklaid F. Stability of maternally reported temperament from infancy to 8 years. Developmental Psychology. 1993;29(6):998–1007.
Language Background	4 years	Parental report	Dichotomised as English speaking background or English as a second language (ESL) if main language spoken to the child is not English	4	

Key to Criteria: 1) cannot be modified through intervention as the factor is biologically driven; 2) comorbid diagnoses such as ADHD, ASD or Learning Disability whose symptoms can be ameliorated to a degree but which cannot be removed entirely; and 4) it would be unethical or impracticable to target in an intervention.

*presented only where criteria for assignment to ‘least mutable’ category is number 3) evidence of limited responsiveness to intervention

^a^ KBIT-2—Kaufman, A. S., and N. L. Kaufman. (2004). *Kaufman Brief Intelligence Test*. *2nd ed*. Bloomington, MN: Pearson;

^b^ATS—Prior, M., Sanson, A., & Oberklaid, F. (1989). The Australian temperament project. In G. Kohnstamm, J. Bates, &M. Rothbart (Eds.), *Temperament in childhood* (pp. 537–554). Chichester: Wiley

#### Mutable-distal factors

For the purposes of this study we defined mutable-distal factors as potentially modifiable factors that exert indirect effects on child language and which, in order to be modified, would require interventions delivered at a population level through social policy rather than through individual families or schools (see Table 2).

**Table 2:** Mutable-distal predictors: measures, age of measurement, and derivation.

Predictor	Age	Measure(s)	Derivation
Social Disadvantage-Socio-economic Indexes for Areas (SEIFA) Index for relative social disadvantage ^a^	8 months	Parental report	Children’s postcodes were used to assign a SEIFA Index. These scores were then assigned a quintile score with reference to Australian SEIFA Quintiles such that a high score represented high levels of disadvantage (Q1 >1054.14 and ≤ 1170.81; Q2 >1013.94 and ≤ 1054.8; Q3 >984.709 and ≤ 1013.94; Q4 >951.247 and ≤ 984–709; Q5 > 0 and ≤ 951.247)
Low Income	8 months	Parental report	Whether or not the family held a healthcare benefit card was used as a proxy measure for low income creating a dichotomous variable. The card is a means tested benefit given to families with a low income.
Maternal age	8 months	Parental report	Dichotomised as young mother (<25 years) or not young mother (≥ 25 years)
Birth Position	8 months	Parental report	
Maternal Education	8 months	Parental report	Dichotomised as low maternal education (last year of school completed < year 12) or not (completing year 12 or above)
Family Literacy	12 months; 4 years	Mill Hill Vocabulary Scale (MHVS)^b^; Wide Ranging Achievement Test 4^th^ Edition (WRAT-4)^c^	A family literacy composite was created by scaling mothers' and fathers' MHVS scores and the WRAT score of the primary carer to a z score (*M* = 0, *SD* = 1), then summing and standardising the summed score (*M* = 0, *SD* = 1).

^a^Australian Bureau of Statistics. (2001). *Socio-Economic Indexes for Areas*. Canberra: Australian Bureau of Statistics.

^b^MHVS Raven, J., Raven, J. C. and Court. J. H. (1998). Manual for Raven’s Progressive Matrices and Vocabulary Scales. Section 5: The Mill Hill Vocabulary Scale. San Antonio, TX: Harcourt Assessment;

^c^WRAT—Wilkinson, G. S., and Robertson, G. J. (2006) *The Wide Range Achievement Test-4th edition* Lutz, FL: Psychological Assessment Resources

#### Mutable-proximal factors

Mutable-proximal factors were defined as those that have the potential to be modified by direct interventions with the child or family and which have robust supporting evidence that they can be substantially ameliorated through intervention (see Table 3).

**Table 3:** Mutable-proximal predictors: measures, age of measurement, derivation and evidence for potential to be modified through intervention.

Predictor	Age	Measure(s)	Derivation	Evidence of responsiveness to intervention
Child Factors				
Conduct Problems score	4 years	The Strengths and Difficulties Questionnaire (SDQ)^a^	Dichotomised as Conduct problems (score ≥ 4) versus no problems (score < 4) (Goodman 1997) equivalent to 90^th^ centile or above.	Hutchings J, Bywater T, Daley D, Gardner F, Whitaker C, Jones K, et al. Parenting intervention in Sure Start services for children at risk of developing conduct disorder: pragmatic randomised controlled trial. British Medical Journal. 2007;334(7595):678–82.
Peer problems score	4 years	SDQ	Dichotomised as Peer problems (score ≥ 4) versus no problems (score < 4) (Goodman 1997) equivalent to 90^th^ centile or above.	Domitrovich CE, Cortes RC, Greenberg MT. Improving young children’s social and emotional competence: A randomized trial of the Preschool PATHS curriculum. Journal of Primary Prevention. 2007;28(2):67–91.
Pro-social behaviour score	4 years	SDQ	Dichotomised as low pro-social behaviour (score ≤ 4) versus typical pro-social score (score > 4 7) (Goodman 1997) equivalent to 10^th^ centile or below.	Sylva K, Melhuish E, Sammons P, Siraj-Blatchford I, Taggart B. Effective Pre-school and Primary Education 3–11 Project (EPPE 3–11) Final report from the primary phase: Pre-school, school and family influences on children’s development during Key Stage 2. Nottingham: DCSF, 2008 DCSF-RR061.
Emotional symptoms score	4 years	SDQ	Dichotomised as Emotional problems (score ≥ 5) versus no problems (score < 5) (Goodman 1997) equivalent to 90^th^ centile or above.	James AC, James G, Cowdrey FA, Soler A, Choke A. Cognitive behavioural therapy for anxiety disorders in children and adolescents. The Cochrane Library. 2013;6:6. Cochrane Database of Systematic Reviews. 2013;6.
Hyperactivity score	4 years	SDQ	Dichotomised as Hyperactivity (score ≥ 7) versus no problems (score < 7) (Goodman 1997) equivalent to 90^th^ centile or above.	Johnston C, Park L. Interventions for Attention-Deficit Hyperactivity Disorder: a year in review. Current Developmental Disorders Reports. 2015;2:38–45.
Speech development	4 years	Goldman Fristoe Test of Articulation (GFTA)^b^	Dichotomized as Speech Sound Disorder (SSD) (GFTA score <10^th^ centile) versus typical score (≥ 10^th^ centile (Goldman and Fristoe 2000)	Law J, Z. G, Nye C. Speech and language therapy interventions for children with primary speech and language delay or disorder. Cochrane Database of Systematic Reviews. 2010(5).
Family Factors				
Frequency reading to child	8 months, 1, 2, 3 and 4 years	Parental report	At each data wave parents were asked how often they read to their child (not very often (1), sometimes (2) or often (3)). The scores over the 5 data waves were averaged and converted to quartiles (Q1. 3 (‘often’); Q2. 2.6–2.8; Q3. >2.2 <2.6; Q4 < = 2.2 (‘sometimes’ or ‘not very often’))	High P, LaGasse L, Becker S, Ahlgren L, Gardner A. Literacy promotion in primary care pediatrics: Can we make a difference? Pediatrics, 105, 927–934. Pediatrics. 2000; 105:927–34.
Number of children's books in the home	2 years	Parental report	Parents were asked to estimate the number of children's books in the home choosing from >30, 20–30, 10–20 or <10.	Golova N, Alario AJ, Vivier PM, Rodriguez M, High P. Literacy promotion for Hispanic families in a primary care setting: a randomized, controlled trial. Pediatrics. 1999; 103:993–7.
TV viewing	4 years	Parental report	Average daily TV viewing was calculated from parents estimates of weekday and weekend viewing (none (0) less than one hour (2) between 1 and 3 hours (3) between 3 and 5 hours (4) 5 hours or more (5)) and converted into quartiles (Q1 < 2.71; Q2 >2.71 < 3; Q3 >3 <3.71; Q4 >3.71 (with Q3 and Q4 representing more than 3 hours per day on average)).	Dennison BA, Russo TJ, Burdick PA, Jenkins PL. An intervention to reduce television viewing by preschool children. Archives of Pediatric Adolescent Medicine. 2004; 158(2):170–6.

^a^SDQ—Goodman, R. (1997). The Strengths and Difficulties Questionnaire: a research note. *Journal of Child Psychology and Psychiatry*, *38*, 581–586;

^b^GFTA—Goldman R, Fristoe M. (2000) *Goldman-Fristoe Test of Articulation 2*. *2nd edn*. Circle Pines, MN: American Guidance Service

### Statistical analysis

We used individual growth trajectory analysis to investigate change in language ability over time using the multilevel model for change [28], which is comprised of two levels.

*Level 1* represents the individual growth model, which specifies the intercept, slope and shape of trajectory for each individual and captures the residual variance, or scatter, of the observed data around the individual’s hypothesised trajectory. In this case the intercept was the child’s scaled language score at age 4 years, the slope was their rate of change in scaled language score between 4 and 7 years, and the shape was hypothesised to be linear, as the three data points available in this data did not allow for specification of curvilinear changes [28].

*Level 2* represents the nature of the associations between predictors and inter-individual differences in the change trajectories. This is comprised of fixed effects and variance components. The fixed effects capture systematic inter-individual differences in intercept and slope according to different values of the predictors. The variance components capture the residual variance in intercept and slope and the covariance between the intercept and slope after controlling for the effect of the predictors. Consideration of fixed effects allow us to uncover the effect of individual predictors on a child’s starting point at 4 years and their rate of progress between 4 and 7 years. Comparison of variance components across models with differing predictors allow us to estimate the degree of variance in intercept and slope explained by those predictors [28]. Models were fitted using maximum likelihood estimation in Stata [38]. The CELF scaled score was the response variable and age in years, centred on 4 years, and corrected for prematurity if necessary, was the unit of time. These models allowed the exploration of inter-individual differences in children’s language scores at 4 years (the intercept) and their rate of change in relative position between 4 and 7 (the slope).

Correlation between candidate predictor variables was examined and preliminary analyses of candidate predictors were conducted and those included in the final multivariate analyses were chosen if preliminary bivariate growth trajectory modelling demonstrated a significant association with language intercept and/or slope at the p< .1 level; and no difficulties of collinearity between predictors arose.

We then fitted 4 models: an unconditional model containing only level-one predictors (the child’s age as a measure of time) and three conditional models that introduced level-two predictors (the ‘risk’ factors). These models were comprised of 1) least mutable factors 2) least mutable and mutable-distal factors and 3) least mutable, mutable-distal and mutable-proximal factors. Differences between these models in terms of the amount of variance in intercept and slope explained, were explored through consideration of the model variance components and also through calculation of pseudo *R* ^*2*^ measure of model fit (Table 1) [28]. This allowed the estimation of the amount of additional variance in intercept and slope explained by the addition of each category of predictors. Consideration of the fixed effects in each model allowed for the estimation of the association between the individual predictors and the children’s language at 4 (the intercept) and the rate of change in relative position between 4 and 7 (slopes), whilst controlling for the effects of the other predictors in the model.

## Results

Table 4 presents comparisons between the three conditional models with respect to their variance components and pseudo *R* ^*2*^ measures of model fit, and between each conditional model and the relevant unconditional model. This allows for the estimation of how much variance in intercept and slope was explained with the addition of each category of predictors: least-mutable, mutable-proximal and mutable-distal.

**Table 4:** Multivariate growth trajectory models 1, 2 and 3: variance components for Mean CELF scaled standard score at 4 years (intercept) Growth rate per year 4–7 years (slope) and Pseudo R^2^.

	Model 1			Model 2			Model 3		
	Least-mutable Factors			Least-mutable and Mutable-Distal			Least-mutable, Mutable-Distal and Mutable-Proximal		
	N = 834			N = 834			N = 763		
	Variance Components		Pseudo R^2^	Variance Components		Pseudo R^2^	Variance Components		Pseudo R^2^
Random Effects	Unconditional Growth Model	Conditional Model		Unconditional Growth Model	Conditional Model		Unconditional Growth Model	Conditional Model	
Intercept	.87	.71	**.34**	.87	.66	**.43**	.85	.62	**.48**
Slope	.11	.09	**.40**	.11	.08	**.44**	.10	.06	**.67**
Covariance intercept and slope	-.18	.02		-.20	.06		-.15	.24	

Table 5 summarises the fixed effects in the models and presents the coefficients, 95% CI and *p* values for each of the predictors in the three conditional models (1) least-mutable predictors 2) least-mutable and mutable-distal and 3) least-mutable, mutable-distal and mutable-proximal). The proportion of the sample falling into each level of the predictors is also shown. The response variable, CELF scaled scores, had a mean of 0 and *SD* of 1. The unit of measurement for the intercept was therefore 1 *SD* and, for the slope *SD* change per year over the 3-year period studied. We can therefore interpret the coefficients as effect sizes, that is, reflecting differences between risks and reference categories in terms of number of *SDs* and for the slope, the numbers of *SD*s lost or gained per year if a child presents with a specific risk.

**Table 5:** Least-mutable and Mutable (proximal and distal) predictors of language growth: multivariate growth trajectory model coefficients Mean CELF scaled standard score at 4 years (intercept) Growth rate per year 4–7 years (slope).

		Model 1		Model 2		Model 3	
		Least-mutable Factors (N = 834)		Least-mutable and Mutable Distal (N = 834)		Least-mutable, Mutable Distal and Mutable Proximal (N = 763)	
Fixed Effects		intercept	slope	intercept	slope	intercept	slope
	%	Coefficient [95% CI]	Coefficient [95% CI]	Coefficient [95% CI]	Coefficient [95% CI]	Coefficient [95% CI]	Coefficient [95% CI]
**‘Least mutable’ predictors**							
*Gender (Ref*: *Female)*	51.5						
Male	48.5	-.06 [-.18, .05]	-.00 [-.03, .03]	-.11* [-.22, .00]	.00 [-.03, .03]	-.09 [-.20, .02]	.00 [-.03, .03]
*Low birth weight (Ref ≥ 2500g)*	96.1						
Low (<2500g)	3.9	-.09 [-.37, .20]	-.14*** [-.22,-.07]	.03 [-.30, .24]	-.14** [-.22,-.07]	-.02 [-.30, .26]	-.09* [-.16,-.01]
*Non-verbal IQ (Ref*: *Q1 (highest))*	12.9						
Q2	23.7	-.20* [-.40,-.01]	-.02 [-.07, .03]	-.12 [-.31, .06]	-.02 [-.07, .03]	-.07 [-.25, .11]	-.04 [-.09, .01]
Q3	21.9	-.48*** [-.68,-.29]	.00 [-.05, .05]	-.35*** [-.54,-.17]	.00 [-.05, .05]	-.27** [-.47,-.08]	-.01 [-.06, .05]
Q4	20.6	-.68*** [-.88,-.49]	-.01 [-.06, .04]	-.55*** [-.74,-.36]	-.02 [-.07, .03]	- .46*** [-.65,-.27]	-.03 [-.08, .03]
Q5 (lowest)	21.0	-1.10*** [-1.30,-.90]	0.06* [.01, .12]	-.92*** [-1.12,-.73]	.06* [.00, .11]	- .78*** [-.98,-.59]	.04 [-.02, .09]
*Family history (Ref*: *no history)*	75.5						
Positive family history	24.5	-.23***[-.36,-.10]	.02 [-.02, .05]	-.16* [-.28,-.03]	.01 [-.02, .05]	-.14* [- .27, .01]	.01 [-.03, .04]
*Developmental disorder (Ref*: *no diagnosis)*	92.6						
Diagnosed developmental disorder	7.4	-.73*** [-.95,-.51]	-.04 [-.10, .02]	-.61*** [-.82 -.40]	-.04 [-.10, .02]	-.52*** [-.74,-.30]	.03 [-.09, .04]
*Shy/Approach-withdrawal (Ref*: *Low approach score)*	80.4						
Shy/High Approach score	19.6	-.18* [-.32,-.04]	.04* [.01, .08]	-.19** [-.33,-.06]	.05* [.01, .09]	-.22** [-.36,-.09]	.03 [-.01, .07]
*Language background (Ref*: *English-Speaking)*	98.0						
English 2^nd^ Language	2.0	-1.5*** [-1.90, -1.02]	.43*** [.32, .54]	-1.3*** [-1.70,-.85]	.43*** [.32, .55]	-1.03*** [-1.5,-.57]	.43*** [.31, .56]
**Mutable-distal predictors**							
*Social disadvantage index (Ref*: *Q1*: *least disadvantaged)*	50.6						
Q2	30.7			-.12§ [-.24, .00]	.01 [-.03, .04]	-0.10§ [-.23, .02]	.01[-.02, .05]
Q3:	8.0			- .29** [-.50, .09]	.00 [-.05, .06]	-.23* [-.44,-.03]	.02 [- .04, .08]
Q4	6.1			-.07 [-.29, .18]	-.00 [-.07, .06]	.04 [-.20, .29]	-.01 [- .08, .05]
Q5 (most disadvantaged)	4.5			-.26§ [-.53, .00]	-.05 [-.02, .12]	-.26§ [-.53,-.00]	.05 [-.02, .12]
*Low Income (Ref*: *does not hold Healthcare benefit card)*	86.1						
Holds Healthcare benefit card	13.9			-.11 [-.28, .05]	-.01 [-.05, .04]	-0.18* [-.34,-.01]	-.00 [-.05, .04]
*Maternal Age (Ref ≥25 years)*	96.6						
Young Mother (<25 years)	3.4			-.19 [-.50, .12]	.03 [-.06, .11]	.11 [-.43 .21]	.03 [-.06, .12]
*Birth Position (Ref <3* ^*rd*^ *)*	84.9						
High birth Position (3^rd^– 5^th^)	15.1			-.34*** [-.50,-.19]	.03 [.01, .07]	-.27** [-.43,-.11]	.02 [-.02, .06]
*Maternal Education (Ref*: *≥ year 12)*	80.7						
Low Maternal Education (< year 12)	19.3			-.14* [-.28, .00]	.03 [-.01, .06]	-.08 [-.22, .06]	.02 [-.02, .06]
*Family Literacy (Ref Q1 highest)*	19.4						
Q2	20.1			-.27** [-.44,-.10]	-.02 [-.07, .02]	.25** [-.42,-.08]	.02 [-.07, .02]
Q3	20.5			-.37*** [-.53,-.20]	.01 [-.03, .06]	.33*** [-.50,-.17]	.02 [-.03, .06]
Q4	20.1			-.34*** [-.51,-.17]	-.03 [-.08, .02]	.27** [-.44,-.10]	.03 [-.08, .02]
Q5 (lowest)	20.1			-.56*** [-.73,-.37]	-.02 [-.07, .03]	.44*** [-.62,-.26]	.01[-.06, .04]
**Mutable-proximal predictors**							
**Child Factors**							
*Conduct score (Ref*: *no problem score)*	90.7						
Conduct problems	9.3					.04 [-.16, .24]	-.04[-.10, .02]
*Peer score (Ref*: *no problem score)*	90.6						
Peer problems	9.5					-.12 [-.31, .07]	.04 [-.02, .09]
*Pro-social score (Ref*: *typical score)*	93.8						
Low pro-social score	6.2					-.23§ [-.45, .01]	.07* [.00, .13]
*Emotional score (Ref*: *no problem score)*	94.0						
Emotional problems	6.0					.20 [-.05, .43]	-.00 [-.07, .07]
*Hyperactivity/inattention (Ref*: *no problem score)*	90.7						
Hyperactivity/inattention problems	9.3					-.09 [-.30, .12]	- .04 [- .10, .02]
*Speech development (Ref*: *typical score)*	95.5						
Speech Sound Disorder	4.5					-.26§ [-.51, .00]	.04 [-.03, .11]
**Family Factors**							
*Frequency reading to child (Ref*: *Q1 highest)*	27						
Q2	20.7					-.15§ [-.30, .01]	.05* [.01, .10]
Q3	31.7					-.21** [-.35,-.06]	.05** [.01, .10]
Q4 (lowest)	20.6					-.38*** [-.56,-.21]	.02 [-.03, .07]
*Number of children’s books in the home (Ref*: *>30)*	70.1						
20–30	16.3					-.20** [-.35,-.05]	.02 [-.02, .07]
10–20	11					-.32** [-.49,-.14]	-.02 [-.08, 0.02]
< 10	7					-.58* [-1.04,-.12]	.11§ [-.02, .24]
*TV viewing per day (Ref Q1 lowest)*	20.0						
Q2	21.3					-0.08 [-.22, .06]	-.03§ [-.07, .01]
Q3	39.2					-0.09 [-.26, .06]	-.04§ [-.08, .01]
Q4	16.7					-0.11 [-.29, .07]	-.07** [-.13,-.03}

Notes: CELF scaled standard scores mean = 0 and SD 0 = 1. Unit of measurement for intercept is 1 SD and for slope is SD change per year. All predictors coded such that higher scores are hypothesised to carry greater risks and reference categories the lowest risk. Key Q = quantile; CELF = Clinical Evaluation of Language Fundamentals; Ref = reference category;

^§^p< .1;

*p < .05;

**p < .01;

***p < .001.

### To what extent are children’s language trajectories fixed by age 4 and is there heterogeneity in children’s rate of progress between 4 and 7 years?

**Fig 1:**
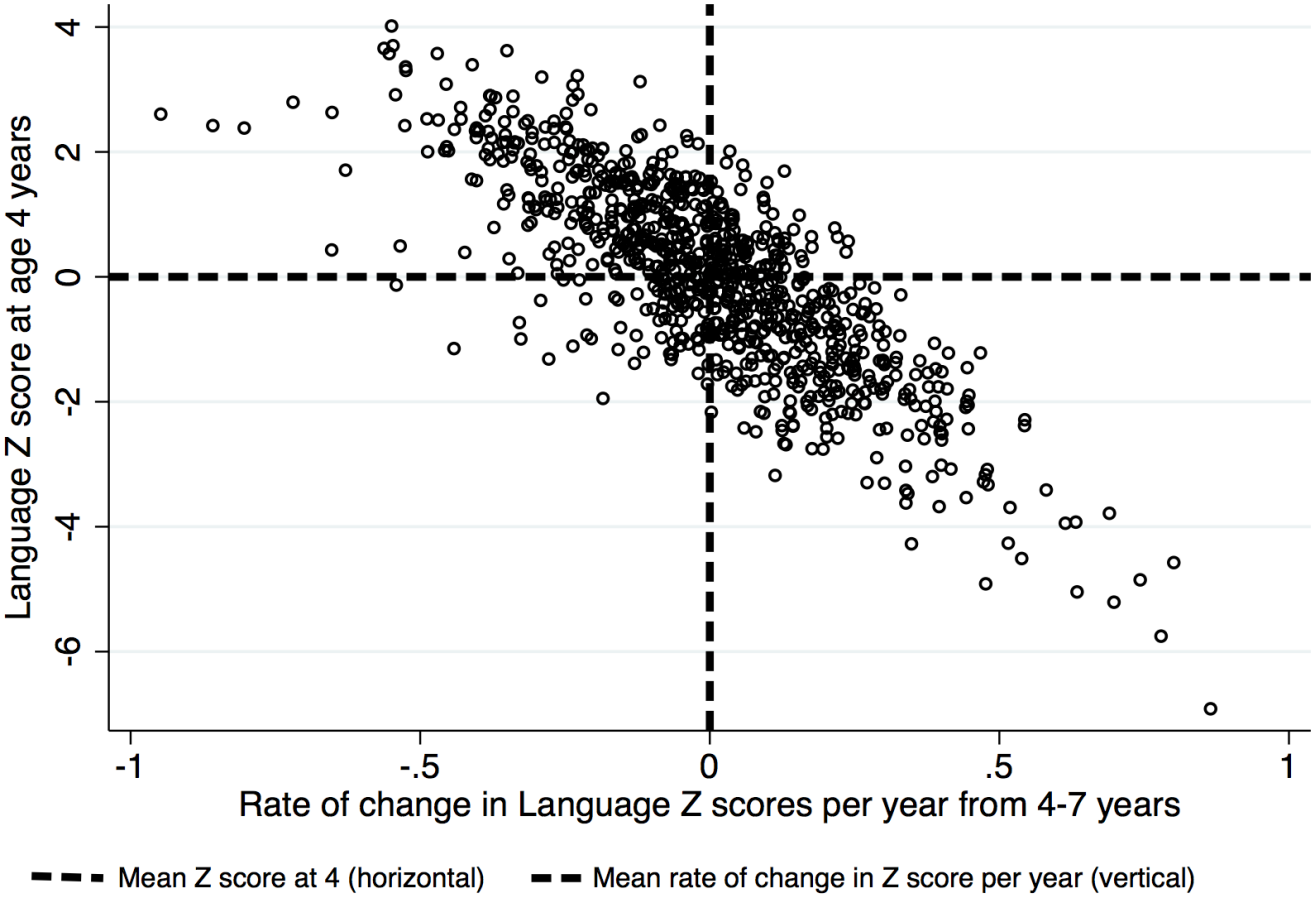
Scatterplot of the relationship between Language Z score at 4 years (intercept) and rate of change in Language Z scores per year from 4–7 years (slope).

The average intercept and slope in scaled CELF score were close to zero in the unconditional model (intercept *M* = .02; slope *M* = .00) reflecting the fact that the language score had been scaled and converted to Z scores. The between person variance around the intercept was .87 and around the slope was .11 (Table 4). This variance in slope indicates a SD of .33. This means that over the 3 years studied, 68% of the sample have rates of progress which vary between approximately +1 and -1 SD per 3 years in language scores and 95% have changes between + 2 and– 2 SD per 3 years. A substantial degree of variability in slope therefore continues to be present in this age range (see Fig 1).

**Fig 2:**
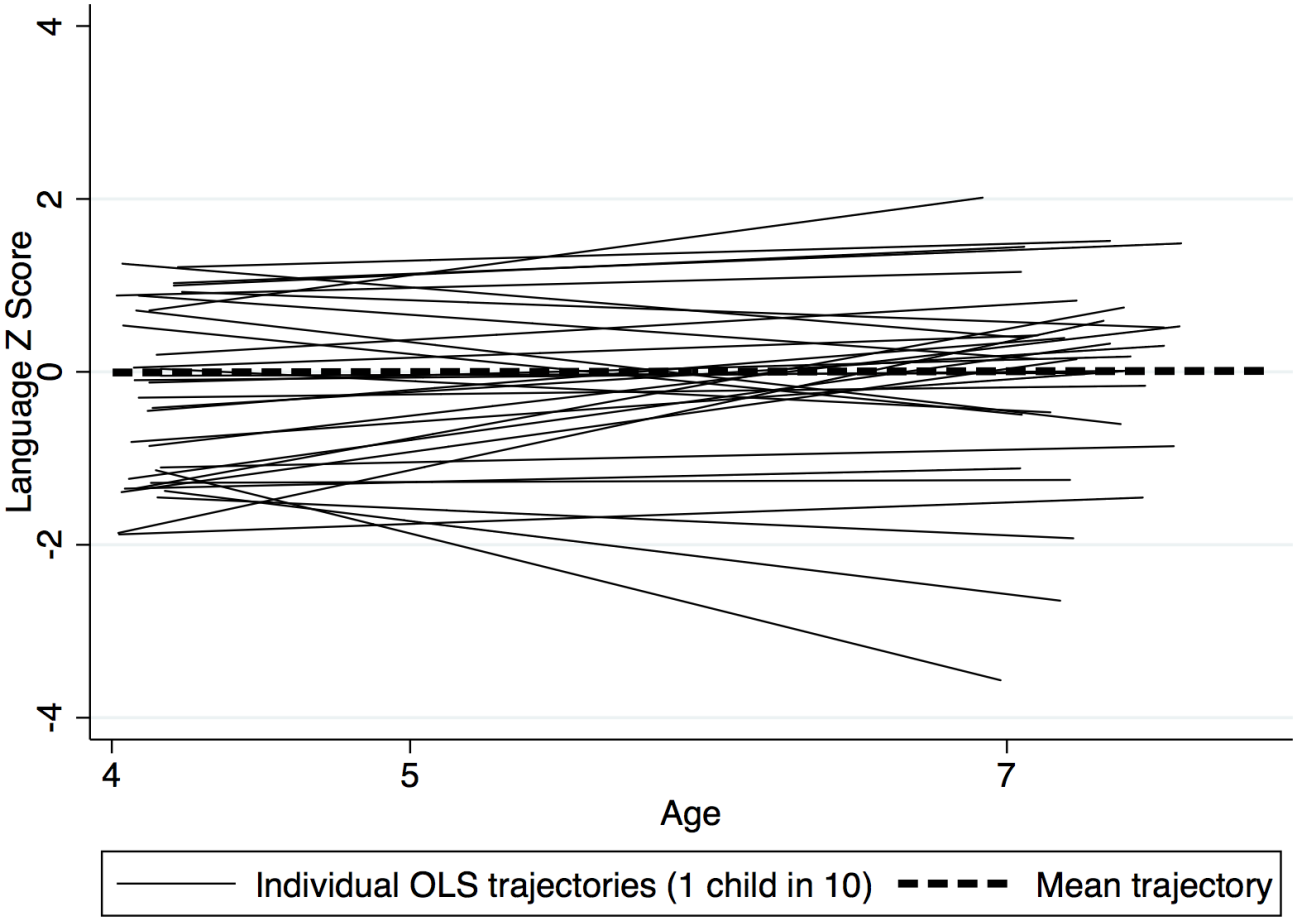
Random sample of ‘empirical growth’ plots—Individual OLS trajectories for 1 child in 10 (N = 83).

**Fig 3:**
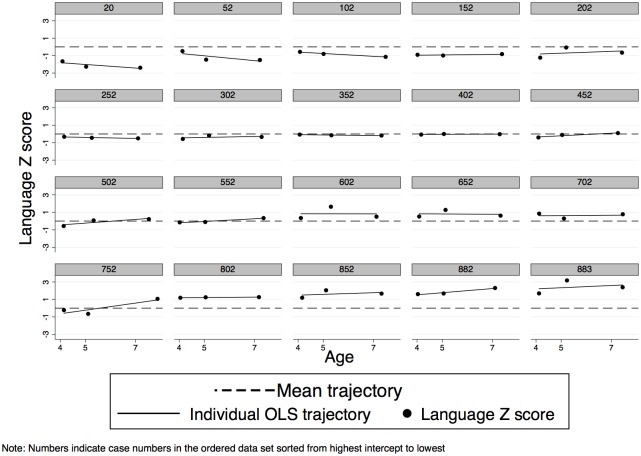
Sample of ‘empirical growth’ plots—sample of 20 individual OLS trajectories ordered from low to high score at 4 years (intercept).

The covariance between intercept and slope was negative suggesting there was a general trend for children with lower scores at intercept to ‘catch up’ and those with higher scores to drop in relative position over time (Table 4 and Fig 1). Figs 2 and 3 depict subsamples of the empirical growth plots (Fig 1 representing 1 child in 10 and Fig 2 representing a sample of 20 children ranked from low to high according to their intercept). These Figures demonstrate the substantial individual differences in intercept and the smaller but significant individual differences in slope that existed in the sample. Furthermore visual inspection of the empirical plots (Fig 3) appears to support the assumption of linearity of our statistical models.

### Which factors predict a child’s language at age 4?

Of the 22 predictors in the final model, 13 made a statistically significant contribution to the variability in the children’s scores at 4 years whilst controlling for the other predictors in the model (Table 5). We note that there was a significant level of correlation between many of the predictors considered (Table 6) which offers explanation for the attenuation of effects seen between Model 1 and Model 3. Significant ‘least-mutable’ *predictors* were: ESL, non-verbal IQ, diagnosis of a developmental disorder, shy/high approach score, and family history of speech and language difficulties. Significant *mutable-distal factors* were family literacy, high birth position, social disadvantage and family income. Significant *mutable-proximal factors* were number of children’s books in the home, and frequency of reading to the child. Having a low prosocial score and a Speech Sound Disorder (SSD) were also significant but only at the *p* < .1 level.

**Table 6:** Correlation between measures examined as predictors of language growth.

Predictor	1:	2:	3:	4:	5:	6:	7:	8:	9:	10:	11:	12:	13:	14:	15:	16:	17:	18:	19:	20:	21:
Correlation																					
p-value																					
1: Gender (Female/Male)																					
2: Low Birth Weight	**-.05**																				
	.007																				
3: Non-verbal IQ (decreasing)	**.09**	**.05**																			
	< .001	.007																			
4: Family history	**.02**	**-.02**	**.05**																		
	.370	.358	.020																		
5: Development Disorder	**.14**	**.08**	**.14**	**.08**																	
	< .001	< .001	< .001	< .001																	
6: Shy Approach	**.03**	**.05**	**.01**	**.00**	**.02**																
	.197	.011	.673	.885	.436																
7: English 2^nd^ Language	**-.04**	**.02**	**-.02**	**.00**	**-.04**	**-.01**															
	.058	.420	.322	.900	.043	.722															
8: Social Disadvantage	**.00**	**.02**	**.12**	**-.03**	**.10**	**.03**	**.09**														
	.811	.211	< .001	.146	< .001	.123	< .001														
9: Low Income	**-.02**	**.00**	**.05**	**.02**	**.02**	**.04**	**.06**	**.12**													
	.223	.820	.008	.254	.209	.070	.001	< .001													
10: Young Mother	**.02**	**-.01**	**.07**	**.02**	**.14**	**-.03**	**.02**	**.15**	**.27**												
	.342	.770	.001	.206	< .001	.125	.289	< .001	< .001												
11: High Birth Position	**-.01**	**.02**	**.10**	**.14**	**.06**	**-.01**	**-.01**	**.00**	**.07**	**-.08**											
	.651	.363	< .001	< .001	.001	.758	.536	.845	.001	< .001											
12: Low Maternal Education.	**-.08**	**-.01**	**.07**	**.05**	**.07**	**.00**	**-.02**	**.19**	**.11**	**.10**	**.04**										
	< .001	.683	.001	.009	< .001	.921	.216	< .001	< .001	< .001	.030										
13: Family Literacy (decreasing)	**-.06**	**.09**	**.10**	**.08**	**.11**	**.01**	**.15**	**.18**	**.13**	**.12**	**.01**	**.20**									
	.003	< .001	< .001	< .001	< .001	.596	< .001	< .001	< .001	< .001	.603	< .001									
14: Conduct Problems	**.09**	**.04**	**.10**	**.04**	**.11**	**-.04**	**-.02**	**.08**	**.05**	**.09**	**.00**	**.04**	**.04**								
	< .001	.052	< .001	.042	< .001	.051	.388	< .001	.007	< .001	.893	.034	.031								
15: Peer Problems	**.04**	**.00**	**.05**	**.09**	**.09**	**.09**	**.04**	**.04**	**.05**	**.07**	**.01**	**.04**	**.00**	**.09**							
	.032	.816	.006	< .001	< .001	< .001	.045	.038	.010	.001	.732	.026	.996	< .001							
16: Low Pro-social Score	**.11**	**.05**	**.06**	**.03**	**.13**	**.02**	**-.04**	**.01**	**.01**	**-.02**	**.05**	**.04**	**.00**	**.16**	**.18**						
	< .001	.018	.003	.103	< .001	.374	.065	.686	.774	.248	.009	.035	.820	< .001	< .001						
17: Emotional Problems	**.07**	**.02**	**.01**	**.02**	**.06**	**.11**	**-.04**	**.03**	**.05**	**.14**	**-.05**	**.03**	**.04**	**.12**	**.10**	**.12**					
	.001	.239	.751	.433	.003	< .001	.068	.092	.009	< .001	.008	.202	.030	< .001	< .001	< .001					
18: Hyperactivity Problems	**.12**	**.08**	**.05**	**.04**	**.18**	**-.03**	**-.02**	**.04**	**.06**	**.09**	**-.02**	**.02**	**.05**	**.36**	**.09**	**.10**	**.15**				
	< .001	< .001	.021	.042	< .001	.150	.388	.040	.001	< .001	.207	.228	.022	< .001	< .001	< .001	< .001				
19: Speech Disorder	**.00**	**.04**	**-.02**	**.09**	**.17**	**.09**	**-.03**	**.04**	**.02**	**-.01**	**.05**	**.05**	**.06**	**-.01**	**.10**	**.08**	**-.03**	**.03**			
	.869	.031	.410	< .001	< .001	< .001	.116	.045	.241	.572	.019	.018	.002	.550	< .001	< .001	.108	.174			
20: Frequency Read (decreasing)	**.02**	**-.01**	**.18**	**.06**	**.11**	**.01**	**.07**	**.08**	**.08**	**.03**	**.23**	**.14**	**.16**	**.04**	**.06**	**.09**	**.04**	**.10**	**.01**		
	.284	.785	< .001	.001	< .001	.659	.001	< .001	< .001	.091	< .001	< .001	< .001	.041	.004	< .001	.044	< .001	.755		
21: Books at Home (decreasing)	**.03**	**-.03**	**.11**	**.05**	**.03**	**-.04**	**.09**	**.08**	**.00**	**.14**	**-.04**	**.12**	**.22**	**.01**	**.08**	**.03**	**.03**	**.02**	**.04**	**.28**	
	.144	.161	< .001	.008	.097	.030	< .001	< .001	.940	< .001	.068	< .001	< .001	.628	< .001	.179	.097	.255	.072	< .001	
22: TV Viewing (increasing)	**.01**	**.03**	**.04**	**.01**	**.04**	**-.04**	**.02**	**.09**	**.08**	**.05**	**.10**	**.08**	**.11**	**.06**	**.07**	**.05**	**.02**	**.05**	**.06**	**.16**	**.14**
	.672	.091	.033	.592	.031	.030	.344	< .001	< .001	.010	< .001	< .001	< .001	.002	.001	.021	.424	.011	.002	< .001	< .001

1: Gender (Female/Male); 2: Low birth weight (≥ 2500g /<2500g); 3: Non-verbal IQ (Quintiles, Q1 (highest)); 4: Positive family history (No/Yes); 5: Diagnosed developmental disorder (No/Yes); 6: Approach score (Low approach score / High-Shy approach score); 7: English 2^nd^ Language (No/Yes); 8: Social disadvantage index (Quintiles, Q1 (least disadvantaged)); 9: Low income (holds Healthcare benefit card) (No/Yes); 10: Young Mother (≥ 25/<25 years); 11: High birth Position (<3^rd^ / 3^rd^–5^th^); 12: Low Maternal Education (≥ year 12/< year 12); 13: Family Literacy (Quintiles, Q1 (highest)); 14: Conduct problems (No/Yes); 15: Peer problems (No/Yes); 16: Low pro-social score (Typical/Low); 17: Emotional problems (No/Yes); 18: Hyperactivity/inattention problems (No/Yes); 19: Speech Sound Disorder (Typical score/ Disorder); 20: Frequency reading to child (Quartiles, Q1 (highest)); 21: Number of children’s books in the home (>30/20-30/10-20/<10); 22: TV viewing per day (Quartiles, Q1 (lowest)).

### Which factors predict a child’s rate of progress between 4 and 7 years?

Six predictors made a statistically significant, unique contribution to the variability in children’s slopes (rate of change between 4 and 7 years) in the final model. Significant *least-mutable* predictors were: ESL, with rapid ‘catch up’ growth evident that was sufficient to make up the disadvantage found at 4 years by 7 years of age; and children with a low birth weight, evincing a small but significant rate of ‘falling behind’ despite having average levels of language at 4 years. The remaining four were *mutable-proximal factors*. The language abilities of the children with low pro-social scores (outside of the typical range for this domain) appeared to ‘catch up’ with their peers over the course of the study. Children with fewer than 10 children’s books in the home, were also catching up. However, at the observed rate of progress, it would be 5 years before they made up their disadvantage in language at age 4. This result was significant at the p< .1 level. Those with 20–30 or 10–20 books did not exhibit catch up growth. Children with the lowest frequency of shared book reading (those whose parents consistently report reading only ‘sometimes’ from 8 months to 4 years) also did not exhibit catch up growth, so their disadvantage at 4 would appear to persist until at least 7 years. Those in the second and third quartile caught up over time, making up their disadvantage over approximately 3 years. Children who watched an average of more than 3 hours TV per day fell behind their peers .07 of a *SD* per year despite the fact that they did not begin with poorer language at age 4.

### How much of the variability in children’s language abilities at 4 and their rate of progress between 4 and 7 years is explained by mutable factors?

Pseudo *R* ^*2*^ values suggest the final model, with all 22 predictors, explains 48% of the variability in intercept and 67% of the variability in slope (rate of progress between 4 and 7 years). The least-mutable factors alone explain 34% of the variability in children’s language abilities at 4 years, mutable distal factors an additional 9% and mutable proximal an additional 5% (Table 4). For the slopes, ‘least mutable’ factors explain 40% of the variability, mutable distal an additional 4% and mutable proximal an additional 23%. Factors which we hypothesised could be changed through interventions therefore explain a large proportion of the variance in slope.

### Multiple imputation

Analyses were completed on participants with complete data for the predictor variables. To test whether the reduced sample size due to missing values of the predictors affected the results, we used multiple imputation to restore the sample to the original 883 children with complete outcome data. There were no substantive differences between growth trajectory analysis results on the imputed and non-imputed data. We concluded that our results could be considered robust.

## Discussion

This paper sought to inform decisions regarding where and when to best focus resources to achieve the greatest gains for children’s language outcomes through an examination of individual differences in language trajectory between 4 and 7 years and of the predictors of those trajectories. Up to the age of 4 individual differences in language abilities were explained, in the most part, by factors that are not suitable targets for interventions such as a child’s gender or a positive family history of speech and language difficulties. However, factors which we hypothesised could be changed through interventions explained a small but significant additional proportion of the variability in language outcomes at 4. Of particular importance up to age 4 years were mutable-distal factors relating to a family’s material and cultural ‘capital’ such as family literacy, high birth position, social disadvantage index and family income [26]. Between the ages of 4 and 7 years individual differences in the rate of language growth were also explained in the most part by the least-mutable group of predictors; in this case ESL and low birth weight. However, a large proportion of variability in growth was also explained by mutable-proximal factors: pro-social scores at 4 years; number of children’s books in the home at 2 years, frequency of shared book reading from 8 months to 4 years and TV viewing at 4 years. There is robust evidence that each of these factors can be modified through interventions with the child and family [39–43] therefore opening up the possibility of early preventative interventions targeting these risks.

This study also demonstrates that children’s relative position in language abilities is not absolutely fixed by age 4 and some movement does still occur between 4 and 7 years, much of which is associated with specific predictors. Our results suggest that the home learning environment up to the age 4 years continues to influence language growth between 4 and 7 years, hence prioritising interventions during the early years of development appear to be supported. These findings support Shonkoff and colleagues’ assertion that “what happens during the first months and years of life matters a lot, not because this period of development provides an indelible blueprint for adult well-being, but because it sets either a sturdy or fragile stage for what follows” [11] p. 5. However, our findings also suggest that substantial changes in a child’s relative position in language ability can still occur after 4 years and that much of this variability remains unexplained, suggesting that scope may still exist to modify children’s language trajectories after the early years. This would suggest that early years interventions should not therefore be prioritised to the exclusion of later interventions.

The effect of the *least-mutable factors*, in the most part, reflected previous research, with children with low non-verbal IQ, diagnosis of a developmental disorder, shy temperament and positive family history all being associated with significantly lower scores at 4 years. Uniquely this study was able to further demonstrate that these risks are not associated with rate of growth between 4 and 7 years.

Although being from a ESL background exerted the highest level of disadvantage on English language scores at age 4, it was also associated with the fastest rate of progress; by the age of 7 this group had caught up with their English-speaking peers. This trajectory was adjusted for other disadvantages, such as low income or high levels of neighbourhood disadvantage. This result would suggest that where poor progress is found for this group of children, the source is unlikely to be their exposure to a minority language at home but rather the broader social risks often experienced by these communities [44].

A further important finding was that, while children with low birth weight did not have lower language scores at 4 years, they experienced a small but significant decline between 4 and 7; a finding which aligns with theories of cumulative vulnerability over time in this group [45]. This result must be interpreted cautiously due to the small numbers with this risk in the cohort however it indicates the importance of the consideration of *growth* in studies of language development as a cross-sectional methodology would not have revealed any differences between this group and their peers.

Factors that could be addressed through social policy such as neighbourhood disadvantage, low income, high birth position and family literacy affected children’s 4-year language scores but were not associated with growth. These factors, which can be broadly conceptualised as a family’s resource, are hypothesised to affect the child indirectly through their effects on proximal processes such as parent-child interaction and the home learning environment [26]. Their importance in the final model, where proximal processes were also included, together with the fact that children do not ‘catch up’ from these disadvantages in the early school years, underline the importance of addressing these more structural inequalities during the very early years.

Factors closest to the child explained a large proportion of the variability in children’s language growth. Counter-intuitively, children with low pro-social scores at 4 years demonstrated catch-up growth in their language abilities between 4 and 7 years. Given that access to good quality early years provision has been shown to have long lasting effects on children’s pro-social scores [39] perhaps access to kindergarten and school addresses these issues for many children and thence their language abilities. More importantly, the child’s home learning environment in the first 4 years of life was associated with children’s language at 4 and had lasting influences into the early school years. The negative effects associated with having fewer children’s books in the home and of low levels of shared book reading in the early years persisted until the age of 7 or, if catch up occurred, would take between 3 and 5 years to be compensated; years during which crucial academic skills are learned. High levels of TV viewing were associated with a negative slope such that children who watched an average of more than 3 hours of TV per day fell behind their peers, perhaps through the displacement of other activities that would benefit language development [46].

When considering the importance of the identified risk factors it is important to remember that many children living with disadvantage are likely to be exposed to multiple risks, thus experiencing cumulative effects [26] which can readily reach clinically significant levels. For example, 5% of this sample was children who were in homes with fewer than 10 children’s books (Coefficient = -.58) *and* had the lowest levels of shared book reading (Coefficient = -.38). Taking account of both of these risk factors this group would, on average, start kindergarten .98 of a *SD* below the mean in language scores, with likely negative consequences extending into adulthood [3,6,7]. Indeed, even children with less severe levels of risk, if aggregated across a number of factors, readily reach similarly poor language levels.

### Limitations and next steps

Due to higher levels of attrition and greater difficulties in recruitment in socially disadvantaged groups this sample does not reflect the more socially disadvantaged groups in the Australian population and so is likely to underestimate the proportion of children at risk. However, as one of the few community samples which include language measures repeated at multiple waves, the cohort provides a rare opportunity to explore *language* growth. The contrast between those factors found in the current study to be associated with language growth and those found by Taylor et al. [18] with respect to vocabulary growth (maternal mental health, school readiness, and socio-economic area disadvantage), would suggest that measures of language development and vocabulary growth tap distinct abilities with distinct risk and protective factors. We therefore urge future cohort studies to include measures of *language* at multiple data waves into adulthood in order to identify more clearly if gaps between children at high and low risk widen, narrow or persist over childhood and to identify risk and protective factors for those trajectories.

The model estimates reported in this study were limited by the measures available in ELVS and the timing of their measurement. For example measures of the quality of childcare/kindergarten provision accessed by the children were not available, nor was detail regarding the school attended by each child. Given the proportion of a child’s day spent in educational provision between 4 and 7 years, we recommend that future work be carried out to determine whether differences exist *between schools* with respect to children’s language growth. Also, although measures of children’s pragmatic skills were available, they were measured after the age of 4 years and so could not be included. The final model, with 22 predictors, is the product of an initial analysis of 36 potential predictors and includes only those factors found to significantly predict language outcomes in a bivariate analyses and which did not present issues with respect to collinearity. The model cannot be entirely comprehensive but we are confident that it captures the majority of relevant factors that the current “state of the science” allows us to measure within a longitudinal cohort design.

It is, of course, essential to note that the direction of causality in observational studies such as this is, inevitably, moot. Hence low language abilities may be the cause of, for example, poor peer relationships rather than the result. Indeed reciprocal developmental relationships between language and a number of the risks identified here are likely (e.g. TV viewing, shared book reading). This study does not prove causality however, given that effective interventions already exist to modify each of the mutable-proximal factors identified as significant in this study, it does clearly signpost potential areas for intervention research. That is to determine whether child language outcomes can be improved through the modification of the associated risks of shared book reading, number of books in the home and TV viewing. It is essential to acknowledge that these risks are likely the most readily measured features of a broader set of responsive and stimulating parenting behaviours. Intervention studies should therefore test the promotion of a wider set of parenting behaviours in the early home environment that may promote children’s language and consider the effect of distal factors, such as family resources, on a family’s ability to provide these experiences.

### Conclusion

Language development is the result of complex interactions between the child’s biological make-up, and their environment [11].Whilst a substantial proportion of the variance in both language at 4 and language growth between 4 and 7 is explained by the factors that are least amenable to change, scope remains to make clinically meaningful differences to children’s language progress through the manipulation of mutable factors. Both proximal factors affecting the home learning environment and those relating to wider social risks were important in creating optimal conditions for the child. The significant and cumulative effects of, shared book reading, books in the home and TV viewing, point to the promotion of a set of parenting behaviours which could bolster language and literacy development, all of which have proven to be modifiable through interventions [39–42]. Preventing or ameliorating literacy difficulties in parents would also appear to be an important goal, with the potential perhaps, to address the intergenerational transmission of language and literacy difficulties [43]. A family’s resource, in terms of its material and cultural capital, which has such a large influence on language development up to the age of 4 years, must not be ignored in the design of interventions [26]. However, the effect of parenting behaviours found over and above other risks, suggest that what parents do with their child also matters.

Preventative interventions in the early years should therefore continue to be a priority and should acknowledge both the quality of the home learning environment and wider social risks. However it is also clear that substantial change can still occur in children’s language growth from 4 to 7 and that much of this growth is associated with mutable factors. Combining interventions for these environmental factors with more traditional, language-focussed approaches could increase the effectiveness of services for children with poor language development. The findings suggest that child language intervention programs should not look for a ‘silver bullet’, rather we should acknowledge the effects of multiple, cascading and cumulative risks and seek to promote child language development through the aggregation of marginal gains in the pre-school years and beyond.
